# Factors associated with total coliform and total viable bacterial count in camel milk from Isiolo County, Kenya

**DOI:** 10.14202/vetworld.2022.1954-1960

**Published:** 2022-08-18

**Authors:** George Karuoya Gitau, Peter Kimeli, Davis Ikiror, Willy Mwangi, Douglas Machuchu, Moses Irungu Gakuru, Genevieve Owuor

**Affiliations:** 1 Department of Clinical Studies, Faculty of Veterinary Medicine, University of Nairobi, P.O. Box 29053-00625 Nairobi, Kenya; 2 Vétérinaires Sans Frontières Suisse, Muthangari Road, off Gitanga Road, P.O. Box 25656-00603, Nairobi, Kenya

**Keywords:** camels, coliform counts, milk, pastoralists, total bacterial count

## Abstract

**Background and Aim:**

Camels have adaptive features to harsh climatic conditions, which make them a valued stable source of livelihood in arid lands. This study estimated the total bacterial and coliform counts (CCs), their associated factors in raw camel milk from the pastoral camel keepers, and the entire milk value chain in Isiolo County and Nairobi, Kenya. This study elucidates the bacterial load in camel milk and its potential risk. Moreover, this study provides recommendations on how to avert a human health hazard.

**Materials and Methods:**

This cross-sectional study targeted the camel pastoralists in Isiolo County, in the northern central part of Kenya. The study was conducted in July and August 2021. In addition, camel milk samples were collected along the value chain key points, including the producers, transporters, one bulker, and small traders in Isiolo town, and other retailers in Nairobi City. Eight camel herds were purposively selected and visited for a sample collection from pooled milking containers (10 mL each). In addition, milk was collected from pooled milk through the transporters, two milk bulkers, and several milk retailers in Isiolo town. Milk was further collected from retailers in Eastleigh town, Nairobi City. At each sampling point, 20 mL of milk sample was collected aseptically. The milk samples were assessed for total viable bacterial counts (TVBC) and CCs using the plate count and digital colony count, respectively.

**Results:**

A total of 76 respondents were selected and 213 milk samples were collected in this study. The respondents included the 1 (1.3%) bulker, 32 (42.1%) producers, 26 (34.2%) traders, and 17 (22.4%) transporters. Most respondents were male (62%), with most being over 40 years old. Overall, the TVBC had a mean of 2436835 ± 9276636 and a median of 3600. Furthermore, the multivariable multilevel mixed-effects linear regression model indicated that gender and practice of smoking milk-handling containers were positively and negatively associated with high counts of the natural logarithm of total viable bacteria, respectively. Of the milk samples evaluated for the CC, 10.3% (22/213) had counts greater than or equal to (≥) 100,000, where some also indicated extreme outliers of about 9.3 million.

**Conclusion:**

This study reports a low proportion of camel milk samples with high total viable bacterial and CCs. The material of the milk container and level of education interactively affected the total viable bacteria.

## Introduction

Africa hosts about 80% of the camel population, which is estimated at 22 million. About 60% of this population is found in the Horns of Africa. It has been estimated that the global camel population has been growing annually at about 3.4% for the last five decades [1]. Kenyan camel milk production has significantly increased following the emergence of new camel keepers in the arid and semi-arid lands (ASALs) [2]. Camels have adaptive features for harsh climatic conditions, making them a valued stablesource of livelihood in arid lands [3]. Camels are also known to be less susceptible to many diseases.

In pastoral communities, camel milk is mainly consumed raw without heat treatment. This is because the pastoralists believe that boiling the milk will result in spoilage and damage to vital elements. However, this practice poses health hazards to humans, either directly or indirectly [4], as some of the bacterial and other pathogenic agents found in camel milk can cause infections in human beings. The camel milk can harbor various microorganisms, including *Staphylococcus*, *Streptococcus*, and coliforms, such as *Escherichia coli*. These organisms can serve as significant sources of infections. There is no known standard for camel milk in Africa, which increases the tendency to use the widely consumed cow milk standard. Most of the camel milk produced is consumed locally (88%) and does not reach the urban markets. They are kept for subsistence use by pastoral communities within the area of production. It has been estimated that about 38% of this milk is consumed, whereas 50% gets spoiled due to the absence of appropriate preservation technologies or storage facilities.

National and global regulations have provided hygiene standards for raw milk from cows and other dairy animals [5]. Furthermore, the most commonly used methods for determining the hygiene of raw milk are the bacterial count and somatic cell count (SCC), which have been defined in the European Union [6, 7]. For example, the bacterial count and SCC in raw cow milk should not exceed 100,000 and 400,000/mL, respectively. The two measures reflect the levels of milk contamination and udder health.

When the milk hygiene standards are very low, this creates a potential hazard for consumers. High microbial contamination in raw milk may be a source of pathogenic microorganisms that cause human and milk-borne diseases [8, 9]. The occurrence of milk-borne diseases is significantly reported in communities consuming raw milk compared with those consuming boiled or pasteurized milk [10]. Some of the bacteria contaminants associated with human disease outbreaks from cow milk in Europe and the USA include *Listeria* *monocytogenes*, *Campylobacter* spp., *Salmonella* spp., and verotoxic *E*. *coli* [11–13].

The study was conducted to estimate the total bacterial and coliform counts (CCs) and their associated factors in raw camel milk from the pastoral camel keepers and the entire milk value chain in Isiolo County and Nairobi, Kenya.

## Materials and Methods

### Ethical approval

The study was approved by Biosafety, Animal Use and Ethical Committee, Faculty of Veterinary Medicine, University of Nairobi (Approval no. FVMBAUEC/2021/295).

### Study period and area

The study was conducted in July and August 2021. Milk samples were mainly collected in Isiolo County and a few samples from traders in Nairobi County. In general, Isiolo County is a typical ASAL area located in the lower north eastern region of Kenya. The county covers an area of about 25,382 km^2^ with a total human population of 268, 000 individuals [3]. The county has ten assembly wards distributed in 3 sub-counties, namely, Isiolo township, Merti, and Garbatulla. The administrative headquarter is in Isiolo town [3]. Geographically, Isiolo County lies between 0° 21’ South and 37° 35’ East and the altitude is between 170 m and 1, 100 m. The rainfall pattern is bimodal, unpredictable, and erratic in distribution. Long rains occur from late March to May, while short rains occur from November to December. The annual average rainfall range is between 350 mm and 600 mm while the mean annual temperature is between 24°C and 30°C. Livestock keeping is the major economic activity in the County and is a key source of livelihood. The main livestock species found in Isiolo County include camel, goats, sheep, cattle, donkeys, and poultry. The camel has recently gained popularity and the shift has been occasioned by resilience of the camel, the increasingly frequent and severe droughts resulting in limited pasture and water resources and new economic opportunities especially increased demand for camel meat and milk. The estimated camel population in Isiolo County is about 39,084 individuals[3].

### Study design and data collection

The study was a cross-sectional study. Camel milk samples were collected at critical points along the value chain. Four critical points that included the producers, transporters, two bulkers, and traders (in Isiolo town and in Nairobi) were identified. Milk samples were then collected. In addition to collecting milk samples, data related to factors that predispose camel milk to microbial contamination along the market chain were collected. This was achieved by administering a closed-ended questionnaire to the relevant respondents at each of the six identified critical points. These data included milking procedures, types of milk containers used for storing and carrying milk, method of cleaning milk containers, source of water for cleaning water containers, and methods of milk preservation, among others.

### Milk sampling

Eight camel herds from different wards of Isiolo County were purposively selected and used for sample collection. Milk was sampled between 5:00 am and 7:00 am through milking containers (10 mL each), and each pooling container was used to carry milk from the herd to the bulkers in Isiolo town. Furthermore, milk was collected from the two milk bulkers in Isiolo town. Specifically, samples were collected from the pooling tank, the residual tank, and the tap from the pooling and residual tanks. For the traders in Isiolo town, milk was collected from retailers who received milk directly from the producers and sold to the consumers. The final collection point was from retailers in Eastleigh town, Nairobi County, who received milk directly from the bulkers in Isiolo town and sold the same to the final consumers in Nairobi City. At each sampling point, 20 mL of milk sample was collected aseptically and immediately kept in cool boxes packed with frozen icepacks, where it awaited transportation to Kenya Dairy Board Laboratories.

### Microbiological analysis

Milk samples were assessed for total viable bacterial counts (TVBC) and CC. Dilutions were selected to ensure that the total number of colonies on a plate fell between 30 and 300 for TVBC and between 15 and 150 for CC [14].

### Total viable bac­terial counts

Camel milk sample was serially diluted by adding 1 mL into 9 mL of Maximum Recovery Diluent until a solution expected to give a plate count of 30–300 was obtained. 1 mL of the sample from a chosen dilution was then placed on the Petri dish with 10–15 mL of molten plate count agar, which was further allowed to solidify for 15 min. Thereafter, the plate was incubated for 48 h at 37°C. Finally, bacterial colonies were counted using the manual count method on the plates after incubation. TVBC was then computed by multiplying the count on the plate by 10 n, in which n stands for the number of consecutive dilutions of the original sample [14].

### Coliform counts

About 1 mL of milk sample serially diluted as 1:10 was transferred into sterile plates. Next, 15 mL of molten violet red bile agar having a temperature of 45°C was added to the milk sample, mixed thoroughly, and allowed to solidify for 5–10 min. Furthermore, the mixture was then overlaid with plating agar to inhibit surface colony formation and then incubated at 37°C for 24 h. Bacterial colonies were then counted using the manual count method on the plates [14].

### Data management and analysis

Data from the questionnaires and laboratory results were entered in Microsoft Excel 2010, from where they were imported into Stata 15.1 software (Stata Corp. LLC, USA) after cleaning. Descriptive analysis was first carried out on the data, which included computing proportions for categorical variables and mean, median, standard deviation, and range for the continuous variables.

A multilevel mixed-effects logistic regression (for the dichotomized total CC; cutoff of 100,000 Colony-forming unit [CFU]/mL) and multilevel mixed-effects linear regression (for the natural logarithm of TVBC) analyses were performed to identify risk factors associated with microbial contamination of milk. In the first step, univariable regression analysis for the predictor variables was fitted into separate models to determine their unconditional associations with the natural logarithm of TVBC and CC. In the second step, multivariable logistic and linear regression analyses were fitted for the univariable associations with p ≤ 0.3. Earlier, correlations between predictor variables were evaluated using pair-wise correlation. The final models for both natural logarithms of total viable bacterial and CC were fitted manually through backward stepwise removal of variables with least statistical significance while retaining variables with p ≤ 0.05. Plausible biological interactions between significant explanatory variables in the final model were also tested [15]. Finally, the area under the receiver operating characteristic curve and PRESS statistics were used to evaluate the overall performance of the models.

## Results

### General respondent demographics and milk-handling characteristics

Tables-1 and 3 show the demographics of the respondents for the animal and milk-handling practices. In summary, 76 respondents and 213 milk samples were selected and sampled in this study. The respondents included 1 (1.3%) bulker, 32 (42.1%) producers, 26 (34.2%) traders, and 17 (22.4%) transporters. Most respondents were male (62%), where most of them were over 40 years. Over 60% of the respondents were illiterate who had not received any form of formal education. In addition, most of the milk considered for this study was sampled before noon and mainly contained in a plastic container with less than 10 L of milk at the time of sampling. Most of the respondents used either warm or hot water, which mainly proceeded from tap water, to clean milk containers, and practice of smoking milk-handling containers. In general, the milk containers and persons handling milk were clean.

**Table-1:** Descriptive statistics of respondent/milk handling characteristics and significance of associations with total coliform counts >100,000.

Variable	Category	Number (%) n = 76	Mean	SD	Median	Range	p-value
Gender	Male	47 (61.8)	117013.1	1078825	3	0–1400000	0.014
	Female	29 (38.2)	334616.3	1458889	18000	0–9300000	
Level of education	None	60 (79.0)	91827.2	711430.1	7	0–9300000	0.845
	Primary	11 (14.5)	637254.2	2696182	80	0–14000000	
	Secondary	3 (4.0)	38006.8	84966.8	1	0–190000
	Tertiary	2 (2.6)	34794.3	53992.4	7300	0–97000
Practice smoking of milk handling containers	No	27 (35.5)	148327.7	300036	5450	0–1600000	0.009
	Yes	49 (64.5)	161343.4	1280912	4	0–14000000	
Temperature of water used to clean the containers	Hot	34 (44.7)	32030.3	86455.8	5	0–440000	0.933
	Warm	32 (42.1)	294723	1676490	9	0–14000000	
	Cold	10 (13.2)	48400.4	132116.6	400	0–670000
Source of water used to clean the containers	Tap	43 (56.6)	166000.8	988870.9	6	0–9300000	0.063
	Borehole	24 (31.6)	189940	1470314	7.5	0–14000000	
	River/dam	9 (11.8)	42164.8	129593	725	0–670000
General cleanliness score of the milk containers	Clean	50 (65.8)	61295.1	191916.6	17	0–1600000	0.143
	Dirty	26 (34.2)	271291.8	1687965	8	0–14000000	
General cleanliness score of the respondent	Clean	47 (61.8)	60925.8	191116.4	22	0–1600000	0.110
	Dirty	29 (38.2)	273867.9	1696448	7	0–14000000	
Material of container	Calabash or wooden	9 (4.2)	39346.1	85303.1	110	0–260000	0.296
	Plastic	199 (93.4)	168144.7	1200593	10	0–14000000	
	Stainless steel	5 (2.4)	6120	7372.0	5500	0–18000
Level in the value chain	Bulker	1 (1.3)	18000	-	-	-	0.003
	Producer	32 (42.1)	118671	1127638	2	0–14000000	
	Trader	26 (34.2)	474313.8	1724084	76000	13–9300000
	Transporter	17 (22.4)	55425.8	166364.4	14.5	0–670000
Time of sampling	Before noon	188 (88.3)	174152	1235007	7	0–14000000	0.861
	After noon	25 (11.7)	44197.5	73609.1	67	0–220000	
Age of the respondent	<40 year	32 (42.1)	239896.3	1596429	23.5	0–14000000	0.222
	≥40 year	44 (57.9)	112100.8	813479.9	5	0–9300000	
Amount of milk from which the sample is drawn	<10 L	126 (59.2)	166058	1264164	8.5	0–14000000	0.478
	≥10 L	87 (40.9)	148531.2	999670.2	13	0–9300000	

**Table-2:** Final multilevel mixed-effects logistic regression model for the total coliform count for the 213-milk sampled from 76 respondents.

Variable	Category	OR	95% CI		p-value

			LCL	UCL	
Gender	Male	Baseline			0.046
	Female	5.35	1.03	27.88	
Practice of smoking of milk handling containers	No	Baseline			0.024
	Yes	0.17	0.04	0.80	

OR=Odds ratio, CI=Confidence interval, LCL=Lower confidence limit, UCL=Upper confidence limit

**Table-3:** Descriptive statistics of respondent/milk handling characteristics and significance of associations with the natural logarithm of total viable bacterial count.

Variable	Category	Number (%) n = 76	Mean	SD	Median	Range	p-value
Gender	Male	47 (61.8)	1200860	6334718	2250	18–46000000	<0.001
	Female	29 (38.2)	7621904	15800000	910000	100–7100000	
Level of education	None	60 (79.0)	2611684	10100000	3100	18–71000000	0.5193
	Primary	11 (14.5)	1139655	1820256	6600	100–6100000	
	Secondary	3 (4.0)	2028520	4456464	18000	2000–10000000
	Tertiary	2 (2.6)	4417667	7433385	240000	13000–13000000
Practice of smoking of milk handling containers	No	27 (35.5)	4224313	10900000	740000	360–52000000	<0.001
	Yes	49 (64.5)	2023546	8851419	2000	18–71000000	
Temperatureof water used to clean the containers	Hot	34 (44.7)	1850521	7004097	2700	18–52000000	0.8527
	Warm	32 (42.1)	1557015	8346078	2700	90–71000000	
	Cold	10 (13.2)	7001492	15100000	20500	100–46000000
Source of water used to clean the containers	Tap	43 (56.6)	3180141	10800000	4500	18–71000000	0.0005
	Borehole	24 (31.6)	419589.8	2312729	2000	45–21000000	
	River/dam	9 (11.8)	6368362	14900000	12500	100–46000000
General cleanliness score of the milk containers	Clean	50 (65.8)	3325321	10300000	12000	89–52000000	0.054
	Dirty	26 (34.2)	1413731	7907485	2000	18–71000000	
General cleanliness score of the respondent	Clean	47 (61.8)	3329147	10200000	9100	90–52000000	0.073
	Dirty	29 (38.2)	1389735	7939651	2350	18–71000000	
Material of container	Calabash or wooden	9 (4.2)	15400000	230000000	48000	360–46000000	0.1133
	Plastic	199 (93.4)	1890917	7924837	3500	18–71000000	
	Stainless steel	5 (2.4)	874128	1599379	42000	640–3700000
Level in the value chain	Bulker	1 (1.3)	3700000				0.0000
	Producer	32 (42.1)	1418360	7085621	1900	18–46000000	
	Trader	26 (34.2)	9762721	17500000	1900000	910–71000000
	Transporter	17 (22.4)	367096.9	878282.4	15000	360–4100000
Time of sampling	Before noon	188 (88.3)	2337905	9616395	2700	18–71000000	0.021
	After noon	25 (11.7)	3180795	6242854	180000	640–25000000	
Age of the respondent	<40 year	32 (42.1)	1696906	6978199	4750	45–46000000	0.058
	≥40 year	44 (57.9)	2864350	10400000	2700	18–71000000	
Amount of milk from which the sample is drawn	<10 L	126 (59.2)	2275652	8730934	2500	18–46000000	0.194
	≥10 L	87 (40.9)	2670273	10100000	9100	18–71000000	

SD=Standard deviation

### Factors associated with total CC >100,000

**Figure-1:**
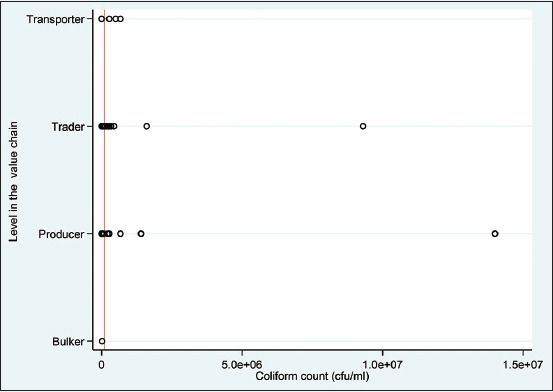
Plot showing the total coliforms counts per level in the value chain for the 213-milk sampled from 76 respondents (red line = cutoff of 100, 000 CFU). CFU=Colony-forming unit.

Of the milk samples evaluated for the CC, 10.3% (22/213) had counts greater than or equal to (≥) 100,000, with some showing extreme outliers of above 9.3 million (Figure-1). Table-2 presents results from the univariable multilevel mixed-effects logistic regression analyses with total CC as a binary outcome variable (1/0 for ≥100,000/<100,000). Some variables that met the p ≤ 0.3 inclusion criteria for the multivariable analyses include gender, practice of smoking milk-handling containers, source of water used to clean the containers, general cleanliness score of the milk containers, general cleanliness score of the respondent, material of container, level in the value chain, and age of the respondent. Furthermore, the multivariable multilevel mixed-effects logistic regression model indicated that gender and practice of smoking milk-handling containers were positively and negatively associated with high (≥100,000) total CCs.

### Factors associated with the natural logarithm of TVBC

**Figure-2:**
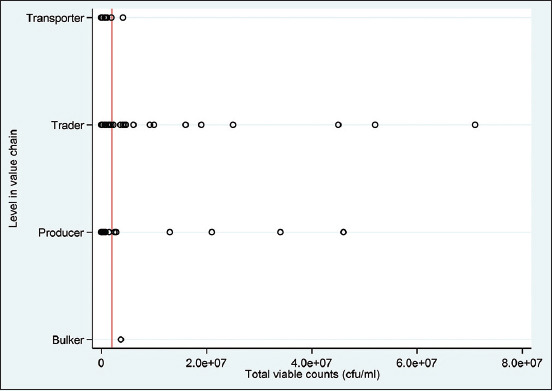
Plot showing the total viable bacterial count per level in the value chain for the 213 milk sampled from 76 respondents (red line = cutoff of 2,000,000 CFU). CFU=Colony-forming unit.

**Figure-3:**
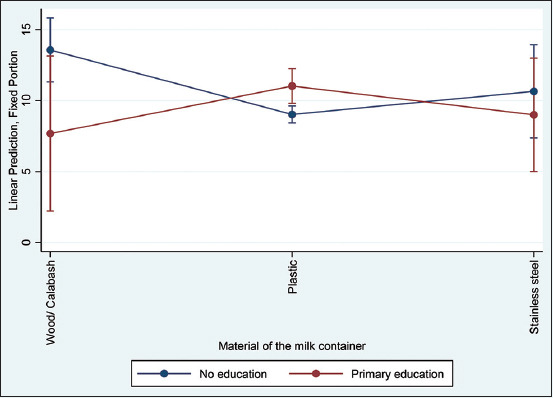
Interaction plot of the predicted natural logarithm of total viable bacterial count and 95% confidence interval for the material of the container and the level of education, based on the final model from 213 milk sampled from 76 respondents.

Overall, the TVBC had a mean of 2436835 ± 9276636, a median of 3600, and a range of 18-71000000. Furthermore, the traders and producers showed the greatest outliers (Figure-2). Table-4 presents results from the multivariable multilevel mixed-effects linear regression analyses with a natural logarithm of the TVBC. The following variables satisfied the p ≤ 0.3 inclusion criteria for the multivariable analyses, namely, gender, practice of smoking milk-handling containers, source of water used to clean the containers, general cleanliness score of the milk containers, general cleanliness score of the respondent, material of the container, level in the value chain, time of sampling, age of the respondent, and amount of milk from which the sample is drawn. Furthermore, gender and practice of smoking milk-handling containers were positively and negatively associated with high counts of the natural logarithm of total viable bacterial, respectively, following a multivariable multilevel mixed-effects linear regression model. The relationship between the container material and the natural logarithm of TVBC also interactively depended on the level of education. In addition, the TVBC was significantly lower for non-educated respondents than for those who had received at least primary education, although this was observed only when the container material was plastic (Figure-3).

**Table-4:** Final multilevel mixed-effects linear regression model for the natural logarithm of total viable bacterial count for the 213-milk sampled from 76 respondents.

Variable	Category	β-coefficient	95% CI		p-value

			LCL	UCL	
Gender	Male	Baseline			<0.001
	Female	3.47	2.27	4.68	
Practice of smoking of milk handling containers	No	Baseline			<0.001
	Yes	−2.86	−4.07	−1.65	
Material of container	Calabash or wooden	Baseline			0.001
	Plastic	−4.54	−6.84	−2.24	
	Stainless steel	−2.91	−6.65	0.82	
Level of education	None	Baseline			0.051
	Primary and above	−5.89	−11.80	0.03	
Material of container* Level of education	Calabash or wooden* None	Baseline			0.023
	Plastic* Primary and above	7.89	1.89	13.88	
	Stainless steel* Primary and above	4.24	−3.06	11.53	

CI=Confidence interval, LCL=Lower confidence limit, UCL=Upper confidence limit

* Interaction term

## Discussion

This study revealed that only 0.9% and 10.3% had TVBC >2 million and CCs greater than 100,000 (CFU/mL), respectively. Therefore, this indicated that more than 90% of the sampled milk was fit for human consumption, considering the guidelines provided by the Kenya Dairy Board (total viable bacterial <2 million CFU/mL; total coliform >100,000 CFU/mL). Reports on the camel milk value chain indicate that only 12% of the milk is marketed, where the bulk of the milk is sold in raw form to rural consumers (10%) and only 2% reaches to urban consumers [16]. Of the remaining milk (88%) that does not reach the market, 38% is directly used by camel-keeping households and their herders as part of their food requirements, while the remaining 50% goes to waste [17]. Therefore, this confirms the need to establish the safety level of the marketed milk.

Our study showed an increased total coliform and TVBCs when the gender of the respondent was female, compared with male. This finding suggests that women could be playing a major role in enhancing the bacterial contamination of camel milk. The previous studies [17, 18] have reported that much time and effort are invested by the pastoral women to maintain their household livelihoods. They engage in other household chores, including collecting water and firewood, grinding maize/sorghum, and looking after children. The poorer the household, the greater the importance of the role of women in ensuring survival and in conducting fundamental productive activities. Consequently, the women exercise more influence over important household decisions [19]. Some duties the women perform expose them to bacterial contaminants, which find their way into the camel milk value chain during milk-handling.

This study revealed that the practice of smoking milk-handling containers significantly reduced total coliform and TVBCs. A previous study conducted by Yiampoi [20] shown that pastoral communities in Kenya use smoke from specific herbs as a technique of disinfecting milk-handling containers, preserving milk, and imparting a characteristic desired flavor to raw camel milk. Smoking is expected to extend the shelf life of camel milk, regardless of high environmental temperatures (>28°C). This traditional technique is known locally by the Isiolo County pastoral communities in Kenya as *qorasum*. During smoking, the containers are inverted over hot smoldering chips until the smoke stops coming out of the container. The residual charcoal pieces are then brushed into the containers with special twigs. Wood smoke contained more than 400 antimicrobial compounds, including acids, alcohols, carbonyls, esters, furans, lactones, and phenols. The compounds in wood smoke originate from the polymers in the wood and the heat-induced chemical reaction between the heated polymers, gasified intermediates, and moisture [19, 20]. Furthermore, most of the compounds in smoke are natural antimicrobials. They are responsible for the preservative effect on milk and their products. They also contribute to the improved organoleptic properties of smoke-treated products [19-21]. This study has demonstrated that smoking of milk-handling containers is effective in hindering microbial growth. Thus, it can be used as a technique for the sanitation and preservation of raw camel milk in arid and semi-arid areas, where cold chains for the preservation of milk are unavailable.

Furthermore, the study’s findings indicated a significant interaction between the container material and the level of education. It revealed that the TVBC was significantly lower for milk sampled from plastic containers among the respondents who did not receive any form of education than those who had primary education. The respondents who had not received any formal education were mostly older; thus, they practiced smoking the milk containers. Moreover, they mostly used plastic containers. The previous studies [19, 22] have shown that milk is commonly handled in plastic containers that are challenged by the difficulty in cleaning them and by the poor quality of water used to clean them, which further enhances the microbial load in milk.

## Conclusion

This study reported that a low proportion of camel milk samples contained high total viable bacterial and CCs. Furthermore, it indicated that gender and the practice of smoking milk-handling containers were positively and negatively associated with high total viable bacterial and CCs, respectively. The material of the milk container and level of education also interactively affected the total viable bacteria.

## Authors’ Contributions

GKG: Designed the study and drafted the manuscript. PK: Collected data and did data analysis. DI: Wrote and proofread the manuscript. WM: Collected data and proofread the manuscript. DM: Designed the study and data collection. MIG: Collected data. GO: Designed the study. All authors have read and approved the final manuscript.
